# Selfishness

**Published:** 1869-02

**Authors:** 


### SELFISHNESS

Is a trait of character universally detested. Some are kind and accommodating to a fault, having a steady lookout for the comfort of others; incommoding themselves that others may be happier. There are many noble names given in history. Bright examples among the living are Miss Dix, Florence Nightingale and multitudes of others, with hearts just as good, whose names may never be known for want of circumstances to bring them before the world.

On the other hand, there are but too many whose hearts never open to another's good, irrespective of any benefit to be derived to themselves; people whose highest, only motive, ever-present, all-pervading, is personal advantage; whose bosoms were never warmed by any one single act of spontaneous kindness; who cannot point to a transaction in the course of a lifetime in which they were actors *for the love of humanity.* As to such the sun rises and sets in self, which is the god of their idolatry. Between characters so beautiful and base, what makes the difference? This is the practical inquiry, and in it all the good are interested, as well as ready to engage in the work of ridding the world of the worse and filling it with the better class.

The main cause of this difference is found in the training of childhood. All Christian people should look the question full in the face and act promptly in reference to it. The petted child is always selfish; the indulged child is always selfish. Such is the natural result of things. If you let your child understand that all his wishes are to be gratified, he must see that in order to do that the wishes of others must be sacrificed in numerous instances. He soon becomes accustomed to this; becomes familiar to its exhibition, and the next step is to claim it as a right, as a matter of course, and soon there is an habitual feeling of irritation whenever the wish is thwarted; an irritation often greater in proportion to the trivial character of the circumstances of the case. Said Lord

Jeffrey, "I am firmly persuaded that indulgence infallibly produces selfishness and hardness of heart."

Let parents take this thing in hand on the instant, and compel their children to habits of practical generosity, of yielding their own wishes and comfort to that of others. Make them familiar with these sacrifices. Soon they will become easy to them, and then they will see their theoretical beauty, and will practice them in the love of them.

This whole subject is broadly illustrated in domestic life in this country. It is exhibited in public assemblies and in all kinds of public conveyances. The contrast between the man and the woman is striking. Many a gentleman in heart and bearing, whose mother and wife and daughter are as blameless in conduct and as beautiful in character as daughter, wife and mother can be, thus predisposing him to see their image in all their sex, must, if he have even ordinary discernment, have his heart pained with the frequent, the very frequent exhibition of a want of courtesy and kindness on the part of women to one another in public places. We see it in the church, in the omnibus, at the concert, on the rail-car, the stage or the steamboat. We have seen a well-dressed woman occupy a double seat in a rail-rar, while gentlemen were allowed to stand, and make room only when specifically requested, and then with the most evident reluctance and ill humor. A daily illustration is presented to the traveller in witnessing a woman making room for a fellow-passenger. She moves as if she felt herself a mountain ; two mountains if it be for one of her own sex. The stolidity with which a woman accepts a seat vacated by a gentleman is a fact of common occurrence.

Within a year we entered a Broadway stage nearly filled. One of the passengers was a poor, woe-begone looking woman, with an arm in a sling, recently broken. Presently two well-dressed young girls entered, and crowded on the sufferer hastily. As she was evidently in pain, and a hasty bodily movement would only increase it, I ventured to remark that the lady's arm was broken.

"Well, if it is, that don't hinder her from moving up."

The utter brutality of such a reply from a well-dressed girl of nineteen may be met with, "*Oh, she was not a lady.*"

We have frequently been an unwilling hearer of coarse abuse of one woman by another in public conveyances for some fancied discourtesy. Observers well know that among men mutual courtesies are the rule; among women, they are the exceptions. All are familiar with the vindictiveness which women exhibit towards their own sex when they become derelict and fall from their high state by the commission of some great crime. It will not answer to say that "these are not ladies of whom you write such things." We know that very well, and that there are millions of women as beautiful in their own characters as those we have described are unlovely. But the inquiry we wish to make here is this, Why should there be on earth a woman so unlovely? Why is it that there are so many? We believe the true answer is, that as a class our daughters are too much indulged by their mothers; their personal ease and personal comfort are too much consulted. As an extravagant but expressive illustration, the mother is at the wash-tub, while the daughter is playing on the piano or the harp; the daughter is asleep, while the mother is cooking her breakfast. It is in the mother's heart that we can always see the angel in promptings and intent; but how misdirected, how injudicious, let any day's exhibition tell. Thus it is that, as a class, daughters are more indulged than sons, and the different results are such as we see daily exhibited. At the private party, in the social visit among her own "set," women are blandness personified, the compromise of her position being before her eyes. Many judge of them as here exhibited, but it is not a fair judgment. Selfishness compels propriety; the advantages are present and certain; but in a crowd of strangers the rewards of courtesy are too remote and too uncertain to warrant a single self-denial. May this not be the key to a large portion of that infelicity which obtains in married life? The daughter transferred from her mother's care, that mother who never spared herself to gratify that daughter's every wish, passes to the new home of her husband, never to be made happy, except by mutual sacrifices and mutual concession. The change is too abrupt; the shock is stunning; the disappointment is crushing. And then follow in the train pouting, peevishness, impatience, chiding, unsparing rebuke; and the man who woke up one morning a few months before and found himself the possessor of an angel, wakes up another morning and finds his angel gone! the only relic being a spoiled child.

Those parents who make the greatest slaves of themselves for the benefit of their children, experience the least heart-comfort from those children in after life; while sons and daughters who have been made to bear their full share of domestic cares and burdens and trials and labors, will grow up with kind hearts and generous natures, making sympathizing husbands, considerate wives, and benevolent citizens.

